# Supplementary material for: Enhancing rainfall estimation accuracy with machine learning, cloud masking, and multi-source data: A case study of four coastal provinces in central Vietnam
Source: PLoS One. 2026 Feb 13;21(2):e0342825. doi: 10.1371/journal.pone.0342825 (PMC12904594; doi:10.1371/journal.pone.0342825)
Supplement: Attachment — Submitted filename: Response to Reviewer.docx [file pone.0342825.s001.docx]

**Responses to reviewers comments**

**Manuscript Title**: **Enhancing Rainfall Estimation Accuracy with Machine Learning, Cloud Masking, and Multi-Source Data: A Case Study of Four Coastal Provinces in Central Vietnam**

**Manuscript ID**: PONE-D-25-46623

Dear Editor and Reviewers,

We would like to express our sincere thanks to the editor and anonymous reviewers for their time spent in evaluating our manuscript and providing us with various constructive comments that help us to improve the presentation of our manuscript. We have carefully addressed all the editor and reviewer’s comments and provided a point-by-point reply. The comments of Reviewers are typeset in normal blue color italic font, and our responses are written in black font color. Additionally, in the revised manuscript, explanations and justifications according to reviewer’s comments have been highlighted in red font color. Other minor corrections have not been highlighted.

Yours sincerely,

Authors.

**1. Response to Editor**

**Point 1:** *Please ensure that your manuscript meets PLOS ONE's style requirements, including those for file naming.*

**Response 1:**

First, we would like to express our sincere gratitude to the Editor for the valuable comments and thoughtful guidance. We have carefully revised the manuscript in accordance with the PLOS ONE style, including adjustments to the title. All corresponding updates and corrections have been incorporated into the revised version.

**Point 2:** *We note that your Data Availability Statement is currently as follows: All relevant data are within the manuscript and its Supporting Information files.*

*Please confirm at this time whether or not your submission contains all raw data required to replicate the results of your study. Authors must share the “minimal data set” for their submission. PLOS defines the minimal data set to consist of the data required to replicate all study findings reported in the article, as well as related metadata and methods (*[*https://journals.plos.org/plosone/s/data-availability#loc-minimal-data-set-definition*](https://journals.plos.org/plosone/s/data-availability#loc-minimal-data-set-definition)*).*

*For example, authors should submit the following data:*

*- The values behind the means, standard deviations and other measures reported;*

*- The values used to build graphs;*

*- The points extracted from images for analysis.*

*Authors do not need to submit their entire data set if only a portion of the data was used in the reported study.*

*If your submission does not contain these data, please either upload them as Supporting Information files or deposit them to a stable, public repository and provide us with the relevant URLs, DOIs, or accession numbers. For a list of recommended repositories, please see* [*https://journals.plos.org/plosone/s/recommended-repositories*](https://journals.plos.org/plosone/s/recommended-repositories)*.*

*If there are ethical or legal restrictions on sharing a de-identified data set, please explain them in detail (e.g., data contain potentially sensitive information, data are owned by a third-party organization, etc.) and who has imposed them (e.g., an ethics committee). Please also provide contact information for a data access committee, ethics committee, or other institutional body to which data requests may be sent. If data are owned by a third party, please indicate how others may request data access.*

**Response 2:**

We appreciate the Editor’s query regarding data availability. The raw meteorological data underlying the results presented in the study are owned by a third party, the National Center for Hydro-Meteorological Forecasting (NCHMF) of Vietnam. These data were provided under a specific license agreement that prohibits public distribution. Therefore, the authors do not have the permission to share the raw dataset publicly. Researchers who meet the criteria for access to confidential data may contact the NCHMF directly to request the data at: www.nchmf.gov.vn (or via email: vtttkttv@mae.gov.vn).

However, to facilitate reproducibility and demonstrate the data structure, we have provided a minimal dataset consisting of processed data from March 2019, September 2019, and June 2020. This dataset is available in the Supporting Information file S1_Minimal_Dataset.zip (code: 10.6084/m9.figshare.30782003).

**Point 3:** *Please amend the manuscript submission data (via Edit Submission) to include author Hung An Nguyen*

**Response 3:**

We have carefully reviewed and thoroughly revised the manuscript during the submission process.

**Point 4:** *Please amend your authorship list in your manuscript file to include author Hung An Nguyen*

**Response 4:**

We have carefully reviewed and thoroughly revised the manuscript in the updated version.

**Point 5:** *We note that Figures 1, 5, 7, 8, 11, and 13 in your submission contain map images which may be copyrighted. All PLOS content is published under the Creative Commons Attribution License (CC BY 4.0), which means that the manuscript, images, and Supporting Information files will be freely available online, and any third party is permitted to access, download, copy, distribute, and use these materials in any way, even commercially, with proper attribution. For these reasons, we cannot publish previously copyrighted maps or satellite images created using proprietary data, such as Google software (Google Maps, Street View, and Earth). For more information, see our copyright guidelines:* [*http://journals.plos.org/plosone/s/licenses-and-copyright*](http://journals.plos.org/plosone/s/licenses-and-copyright)*.*

*We require you to either (a) present written permission from the copyright holder to publish these figures specifically under the CC BY 4.0 license, or (b) remove the figures from your submission:*

*a. You may seek permission from the original copyright holder of Figures 1, 5, 7, 8, 11, and 13 to publish the content specifically under the CC BY 4.0 license.*

*We recommend that you contact the original copyright holder with the Content Permission Form (*[*http://journals.plos.org/plosone/s/file?id=7c09/content-permission-form.pdf*](http://journals.plos.org/plosone/s/file?id=7c09/content-permission-form.pdf)*) and the following text:*

*“I request permission for the open-access journal PLOS ONE to publish XXX under the Creative Commons Attribution License (CCAL) CC BY 4.0 (*[*http://creativecommons.org/licenses/by/4.0/*](http://creativecommons.org/licenses/by/4.0/)*). Please be aware that this license allows unrestricted use and distribution, even commercially, by third parties. Please reply and provide explicit written permission to publish XXX under a CC BY license and complete the attached form.”*

*Please upload the completed Content Permission Form or other proof of granted permissions as an ""Other"" file with your submission.*

*In the figure caption of the copyrighted figure, please include the following text: “Reprinted from [ref] under a CC BY license, with permission from [name of publisher], original copyright [original copyright year].”*

*b. If you are unable to obtain permission from the original copyright holder to publish these figures under the CC BY 4.0 license or if the copyright holder’s requirements are incompatible with the CC BY 4.0 license, please either i) remove the figure or ii) supply a replacement figure that complies with the CC BY 4.0 license. Please check copyright information on all replacement figures and update the figure caption with source information. If applicable, please specify in the figure caption text when a figure is similar but not identical to the original image and is therefore for illustrative purposes only.*

*The following resources for replacing copyrighted map figures may be helpful: USGS National Map Viewer (public domain):* [*http://viewer.nationalmap.gov/viewer/*](http://viewer.nationalmap.gov/viewer/)

*The Gateway to Astronaut Photography of Earth (public domain):* [*http://eol.jsc.nasa.gov/sseop/clickmap/*](http://eol.jsc.nasa.gov/sseop/clickmap/)

*Maps at the CIA (public domain):* [*https://www.cia.gov/library/publications/the-world-factbook/index.html*](https://www.cia.gov/library/publications/the-world-factbook/index.html) *and* [*https://www.cia.gov/library/publications/cia-maps-publications/index.html*](https://www.cia.gov/library/publications/cia-maps-publications/index.html)

*NASA Earth Observatory (public domain):* [*http://earthobservatory.nasa.gov/*](http://earthobservatory.nasa.gov/)

*Landsat:* [*http://landsat.visibleearth.nasa.gov/*](http://landsat.visibleearth.nasa.gov/)

*USGS EROS (Earth Resources Observatory and Science (EROS) Center) (public domain):* [*http://eros.usgs.gov/#*](http://eros.usgs.gov/)

*Natural Earth (public domain):* [*http://www.naturalearthdata.com/*](http://track.editorialmanager.com/CL0/http:%2F%2Fwww.naturalearthdata.com%2F/1/010f0199799ad5d4-a7d23688-8357-4612-b900-fb756fb6b3a7-000000/hJdhVokxW4vuUwG1iLgka5nKi_g2IzpPoIP2ov6XczA=229)

**Response 5:**

We appreciate the Editor’s remarks regarding the copyright of the maps used in our manuscript. We confirm that all maps presented in the paper were entirely created by our research team based on the appropriate specifications, and none were copied from any organizations or individuals. We take full responsibility for the accuracy and originality of these maps.

**Point 6:** *If the reviewer comments include a recommendation to cite specific previously published works, please review and evaluate these publications to determine whether they are relevant and should be cited. There is no requirement to cite these works unless the editor has indicated otherwise.*

**Response 6:**

We sincerely appreciate the Editor’s valuable comments. We have carefully reviewed and thoroughly addressed all the reviewers’ requests.

**Point 7:** *Additional Editor Comments:* *Two reviewers have provided constructive comments to help the authors improve their manuscript. I agree with their suggestions, particularly the need to extend the study period and to conduct a more comprehensive review of the subject matter.*

**Response 7:**

We sincerely appreciate the Editor’s comments. We have carefully considered and addressed the suggestions provided by both the reviewers and the Editor. In response, we have extended the study period from 2 years (2019–2020) to 5 years (2019–2023) to enable a more comprehensive evaluation of the subject matter.

**2. Response to Reviewer A**

**Point 1:** *The model is trained on data from only two years (2019-2020), so from a statistical point of view the period is not relevant. A longer time series is needed, which includes more years with different climatic conditions.*

**Response 1:**

We sincerely appreciate the reviewer’s valuable comment. We fully agree that a training dataset limited to two years (2019–2020) may not be sufficient to capture the diverse meteorological patterns and climatic variability of the region. Therefore, to enhance the reliability and generalization capability of the model, we extended the study period to five consecutive years (2019–2023). The rain gauge and weather radar datasets used in this study are proprietary datasets provided by the National Center for Hydro - Meteorological Forecasting (NCHMF) for the five-year period from 2019 to 2023. Consequently, we were unable to obtain a 10-year dataset as suggested by the reviewer.

However, the classification and rainfall estimation performance of the proposed rainfall product and the comparative rainfall products did not differ significantly when the five-year dataset was used compared to the two-year dataset. The detailed results are as follows:

Specifically, using the two-year dataset, the classification results of the proposed product and the compared rainfall products were as follows:

|  | **CSI** | **ETS** | **POD** | **FAR** | **BIAS** | **HSS** |
| --- | --- | --- | --- | --- | --- | --- |
| Radar | 0.47 | 0.39 | 0.5 | 0.11 | 0.56 | 0.56 |
| IMERG Final Run | 0.35 | 0.24 | 0.43 | 0.34 | 0.64 | 0.39 |
| IMERG Early Run | 0.32 | 0.22 | 0.38 | 0.34 | 0.58 | 0.36 |
| GSMaP_MVK_Gauge | 0.46 | 0.33 | 0.59 | 0.33 | 0.87 | 0.5 |
| PERSIANN_CCS | 0.2 | 0.14 | 0.22 | 0.28 | 0.31 | 0.24 |
| LGBM-3SC-CF | **0.57** | **0.45** | **0.78** | **0.32** | **1.14** | **0.62** |
| LGBM-3S | 0.53 | 0.38 | 0.87 | 0.42 | 1.52 | 0.55 |
| range | [0: 1] | [-1/3: 1] | [0: 1] | [0: 1] | [0: inf] | [-inf: 1] |
| optimal | 1 | 1 | 1 | 0 | 1 | 1 |

In contrast, using the five-year dataset, the results were as follows:

|  | **CSI** | **ETS** | **POD** | **FAR** | **BIAS** | **HSS** |
| --- | --- | --- | --- | --- | --- | --- |
| Radar | 0.51 | 0.40 | 0.54 | 0.15 | 0.63 | 0.58 |
| IMERG Final Run | 0.36 | 0.24 | 0.46 | 0.37 | 0.73 | 0.39 |
| IMERG Early Run | 0.32 | 0.21 | 0.39 | 0.37 | 0.62 | 0.34 |
| GSMaP_MVK_Gauge | 0.48 | 0.34 | 0.74 | 0.40 | 1.24 | 0.51 |
| PERSIANN_CCS | 0.19 | 0.12 | 0.21 | 0.30 | 0.30 | 0.22 |
| LGBM-3SC-CF | **0.55** | **0.43** | **0.74** | **0.30** | **1.04** | **0.60** |
| LGBM-3S | 0.56 | 0.42 | 0.82 | 0.36 | 1.28 | 0.59 |
| range | [0: 1] | [-1/3: 1] | [0: 1] | [0: 1] | [0: inf] | [-inf: 1] |
| optimal | 1 | 1 | 1 | 0 | 1 | 1 |

When evaluated on the extended 5-year dataset, the proposed LightGBM-based rainfall classification product, LGBM-3SC-CF, confirms its superior classification quality compared to other rainfall products. The evaluation metrics demonstrate a remarkable consistency with the results from the initial 2-year dataset, strongly indicating the model's stability and robustness over a longer period. Specifically, while the transition to the 5-year dataset introduced minor declines in several skill and classification success metrics—CSI dropped by 0.02 (from 0.57 to 0.55), ETS by 0.02 (from 0.45 to 0.43), POD by 0.04 (from 0.78 to 0.74), and HSS by 0.02 (from 0.62 to 0.60)—these were compensated by significant improvements in prediction tendency and efficiency. Most notably, the model successfully reduced the False Alarm Ratio (FAR) by 0.02 (from 0.32 to 0.30) and achieved a substantial improvement in the BIAS score, correcting by 0.1 (moving from an over-prediction bias of 1.14 to a better-calibrated value of 1.04). This balanced performance underscores the LGBM-3SC-CF product's ability to maintain high predictive capability and achieve better overall calibration across a more extensive and diverse time series.

For the regression results, using the two-year dataset, the proposed product and the comparative rainfall products in the study area yielded the following outcomes:

|  | **CC** | **MAE** | **RMSE** | **mKGE** | **BIAS** |
| --- | --- | --- | --- | --- | --- |
| Radar | 0.71 | 1.84 | 4.44 | 0.4 | 0.6 |
| IMERG Final Run | 0.46 | 3.55 | 7.70 | 0.39 | 1.11 |
| IMERG Early Run | 0.38 | 3.21 | 6.77 | 0.23 | 0.75 |
| GSMaP_MVK_Gauge | 0.31 | 3.03 | 6.92 | 0.15 | 0.69 |
| PERSIANN_CCS | 0.33 | 3.06 | 6.29 | -0.07 | 0.45 |
| LGBM-3SC-CF | **0.49** | **2.90** | **5.68** | **0.46** | **1.04** |
| LGBM-3S | 0.47 | 3.10 | 5.82 | 0.40 | 1.16 |
| range | [-1: 1] | [0: inf] | [0: inf] | [0: 1] | [0: inf] |
| optimal | 1 | 0 | 0 | 1 | 1 |

Similarly, using the five-year dataset, the proposed product and the reference products obtained the following results:

|  | **CC** | **MAE** | **RMSE** | **mKGE** | **BIAS** |
| --- | --- | --- | --- | --- | --- |
| Radar | 0.70 | 1.78 | 4.33 | 0.46 | 0.65 |
| IMERG Final Run | 0.44 | 3.60 | 7.45 | 0.40 | 1.21 |
| IMERG Early Run | 0.36 | 3.17 | 6.62 | 0.28 | 0.78 |
| GSMaP_MVK_Gauge | 0.44 | 2.49 | 6.52 | 0.32 | 0.61 |
| PERSIANN_CCS | 0.30 | 2.88 | 6.21 | -0.13 | 0.38 |
| LGBM-3SC-CF | **0.47** | **2.66** | **5.48** | **0.47** | **1.09** |
| LGBM-3S | 0.47 | 3.83 | 5.64 | 0.44 | 0.94 |
| range | [-1: 1] | [0: inf] | [0: inf] | [0: 1] | [0: inf] |
| optimal | 1 | 0 | 0 | 1 | 1 |

Evaluation of the rainfall products on the extended 5-year dataset confirms that the proposed LGBM-3SC-CF model maintains its competitive standing. The model remains superior to all other competing rainfall products, only underperforming the benchmark radar product in three specific regression metrics: CC, MAE, and RMSE. Crucially, the LGBM-3SC-CF product scores better than the radar in mKGE and BIAS.When comparing these results to the initial 2-year regression analysis, the model demonstrates high stability, despite minor fluctuations. A slight decrease was observed in the CC (from 0.49 to 0.47) and BIAS (moving from 1.04 to 1.09). However, this was compensated by noteworthy improvements in key error metrics: MAE decreased significantly (from 2.90 to 2.66), RMSE also decreased (from 5.68 to 5.48), and mKGE improved slightly (from 0.46 to 0.47). Meanwhile, the other rainfall products generally maintained stable performance, with the sole exception of the GSMaP_MVK_Gauge product, which exhibited significant improvement across its indices. This performance underscores the robustness and effective error reduction capability of the LGBM-3SC-CF model over a longer, more diverse time series.

In addition, the FY-4A product has been removed from the revised manuscript due to the lack of consistent data coverage over the entire five-year period. The updates related to the dataset duration have been incorporated into **Section 2.2 Datasets**. Furthermore, the total annual rainfall for the years 2019–2023 has been summarized in Figure 2 (**Section 2.2 Datasets**, and the number of rainfall samples included in the input datasets of the classification models M1, M2, M3, and M4 has been recalculated and presented in Figure 4 (**Section 2.3.4 Training and Evaluation**).

**Point 2*:*** *The use of cloud masks, while reducing false alarms, also led to a decrease in the probability of detection, and with regard to extreme events the model cannot be considered relevant, an aspect also recognized by the authors.*

**Response 2:**

We appreciate the reviewer’s accurate observation regarding the trade-off inherent in our approach. In this study, we utilized cloud masks (CM1 and CM2) as strict conditional constraints to define areas of potential rainfall (Model M1) and high-intensity rainfall (Model M2).

Specifically, a pixel is classified as 'rain' only if it is simultaneously identified as cloudy by CM1 and is classified as rain by M1. While this strict criterion may reduce the Probability of Detection (POD), it effectively lowers the False Alarm Ratio (FAR) and increases the Critical Success Index (CSI) by minimizing false positives. This strategy is crucial for handling the severe class imbalance in the dataset, where no-rain samples significantly outnumber rain samples.

Similarly, for intensity classification, a pixel is designated as 'high-intensity rain' only if it satisfies both the CM2 condition (high-intensity rain cloud) and the M2 model classification of high-intensity rain. While we acknowledge that this approach might miss some extreme rainfall events (which are often localized and rare), it significantly improves overall estimation accuracy because actual rainfall events are predominantly low-intensity. By mitigating the misclassification of low-intensity rain as high-intensity, the model achieves lower MAE, RMSE, and BIAS in the rainfall estimation tasks.

In summary, while the use of cloud masks may limit the precise detection of rare extreme rainfall events, it significantly enhances the reliability and overall accuracy of the rainfall product under the majority of weather conditions. Furthermore, our proposed cloud masking approach is particularly well-suited for rainfall classification model training scenarios characterized by severe class imbalance in the input datasets.

**Point 3:** *The use of rain gauge data for supervised machine learning training makes the accuracy of the model good but is related to their resolution.*

**Response 3:**

We fully agree with the reviewer’s insightful comment. Rain gauge observations are considered the “ground truth” and generally provide higher accuracy than other remote sensing measurements such as radar or satellite derived precipitation estimates. We also acknowledge that spatial resolution (or density) can indeed influence the accuracy of rainfall estimation, particularly when assessments are conducted over larger regional scales. However, within the scope of this study, we used observations from 175 rain gauge stations provided by the National Center for Hydro - Meteorological Forecasting (NCHMF) of Vietnam, which are relatively well distributed across the entire study area. This spatial arrangement ensures adequate spatial representativeness for the study region.

Figure 1. Distribution of rain gauge stations in the study area.

**3. Response to Reviewer B**

**Point1:** *The abstract, for example, contains an excessive amount of detail that obscures the main purpose and contribution of the study. It should be thoroughly revised to be more concise and focused, presenting only the essential elements: the research problem, objectives, a brief description of the proposed approach, the main results, and their significance. Overly detailed methodological explanations and lengthy background information should be removed to ensure that the abstract clearly conveys the essence and relevance of the study to a broad audience.*

**Response 1:**

We sincerely thank the reviewer for the precise and constructive comment regarding the length and clarity of the Abstract. We have carefully reviewed and revised the content to make it more concise and succinct. The revisions are highlighted in blue text in the revised manuscript.

***Revised Abstract****:* “This study develops the LGBM-3SC-CF product, a machine learning framework that integrates multi-source data (Himawari-8, ERA5, ASTERDEM, and rain gauge) to significantly improve rainfall classification and estimation accuracy for four coastal provinces in Central Vietnam. Methodologically, our core contribution lies in the proposed 3-stage classification architecture combined with a novel cloud masking technique. To address the severe class imbalance inherent in rainfall datasets, we further employed an effective data balancing technique based on rainfall intensity distribution alongside feature selection based on cloud- and rain-forming factors. The performance of the proposed rainfall product was compared with four existing regional rainfall products: IMERG Final Run V07, IMERG Early Run V06, GSMaP_MVK_Gauge V07, and PERSIANN-CCS. The LGBM-3SC-CF achieved the highest performance, with a Critical Success Index (CSI) of 0.55 and a Probability of Detection (POD) of 0.74, and achieved the Correlation Coefficient (CC) of 0.47 and the Modified Kling-Gupta Efficiency (mKGE) of 0.46. Furthermore, it obtained the lowest error metrics, with a Mean Absolute Error (MAE) of 2.66 mm/h and a Root Mean Squared Error (RMSE) of 5.48 mm/h. This study not only establishes a robust machine learning framework but also provides the essential methodological foundation for developing near real-time rainfall estimation models through the seamless substitution of the atmospheric reanalysis features with near real-time meteorological features.

**Point 2:** *Similarly, the introduction does not adequately place the study in the context of existing research. It lacks a critical synthesis of previous studies and does not clearly articulate the specific knowledge gaps that the work seeks to address. To strengthen this section, the author should integrate the relevant literature more effectively, highlight the limitations of previous work, and explicitly explain how this study builds on and advances previous efforts. A more thorough and focused literature review would make the novelty and research needs clearer to the reader.*

**Response 2:**

We sincerely thank the reviewer for the accurate and constructive comment. We have carefully considered this feedback and rigorously revised **Section 1. Introduction** to ensure scientific coherence. Specifically, we have clarified the limitations of previous studies to better underscore the novel contributions of our research into this section in the revised manuscript.

**Point 3:**  *The methodology section also needs significant reorganization. Its current structure is fragmented, and its logical sequence is difficult to follow. Authors are encouraged to begin by outlining the overall methodological framework and data processing steps before explaining the theoretical and algorithmic details of the LightGBM model. Establishing a clearer structure will help the reader understand the study design and the rationale for the chosen approach.*

**Response 3:**

We sincerely thank the reviewer’s constructive suggestion to improve the logical flow of the Methodology section. We have revised the layout by prioritizing the general framework and data processing steps before detailing the specific algorithms. The section has been reorganized from the original sequence (**Model Architecture**, **Algorithms**, **Cloud Mask Generation**) to the revised order: **2.3.1 Framework**, **2.3.2 Cloud Mask Generation**, **2.3.3 Algorithms**, followed by **2.3.4 Training and Evaluation**.

**Point 4**: *A further limitation is the exclusive reliance on the LightGBM model without comparison with other robust machine learning or statistical models. Without such a benchmark, it is difficult to assess the relative performance or originality of the proposed method. Incorporating additional underlying models such as Random Forest, XGBoost, Support Vector Machines, or appropriate deep learning models would provide a stronger basis for evaluating the effectiveness and specificity of the approach.*

**Response 4:**

Thank you for your comment.

We sincerely thank the reviewer for the insightful and constructive comments. We fully agree that examining solely the LGBM algorithm was insufficient to adequately assess its relative performance or uniqueness within the proposed framework for rainfall classification and estimation.

Consequently, we have expanded our investigation to include two additional machine learning algorithms: Random Forest (RF) and XGBoost (XGB). This comparative analysis aims to identify the most effective algorithm for both classification and regression tasks. These updates have been incorporated throughout the manuscript, with detailed descriptions of these algorithms added to **Section 2.3.3 Algorithms.**

**Point 5:** *In addition, the manuscript does not describe any model optimization or hyperparameter tuning strategies. Since hyperparameter tuning is essential to achieve robust and reproducible performance, authors should clearly state the optimization method used (such as grid search, random search, Bayesian optimization or Optuna) and report the tuned parameters used in the final model. Providing this information is essential to ensure transparency, reproducibility and fairness in model evaluation.*

**Response 5:**

Thank you for your comment.

We fully agree with the reviewer’s comment and acknowledge that the previous manuscript lacked a detailed description of the model optimization and parameter tuning process. To address this omission, we have revised the manuscript as follows:

We employed the Grid Search technique to investigate and select optimal values for the set of hyperparameters, including: n_estimators, max_depth, subsample, colsample_bytree, class_weight, and learning_rate. The surveyed range for each parameter and the selected optimal values are summarized in Table 7.

*Table 7.* *Hyperparameter optimization*

| **Hyperparameter** | | **Optimal value** | | | | | | | |
| --- | --- | --- | --- | --- | --- | --- | --- | --- | --- |
| **Name** | **Examined values** | **M1** | **M2** | **M3** | **M4** | **M5** | **M6** | **M7** | **M8** |
| n_estimators | 500; 700; 900; 1500; 2000; 2500 | 2000 | 2000 | 1500 | 1500 | 1500 | 900 | 1500 | 700 |
| max_depth | 8; 12; 14; 16; 18; 24; 28; 32, 36 | 28 | 28 | 24 | 24 | 24 | 18 | 18 | 16 |
| subsample | 0.6; 0.8; 1.0 | 0.8 | 0.8 | 0.8 | 0.8 | 0.8 | 0.8 | 0.8 | 0.8 |
| colsample_bytree | 0.6; 0.8; 1.0 | 0.8 | 0.8 | 0.8 | 0.8 | 0.8 | 0.8 | 0.8 | 0.8 |
| Class weight | 1.6; 1.8; 2.0; 2.5; 3.0; 5.5; 7.5; 9.5; 10.5; 12.5 |  | 1.8 | 1.6 | 9.5 |  |  |  |  |
| learning_rate | 0.1; 0.01; 0.001; 0.002; 0.005, 0.08 | 0.01 | 0.01 | 0.01 | 0.01 | 0.005 | 0.005 | 0.002 | 0.002 |

Accordingly, we have added a new section, section **3.1.3 Hyperparameter Optimization**, which presents a detailed breakdown of the surveyed values and the optimal hyperparameters selected for both the rainfall classification and regression models.

**Point 6:** *The discussion section would also benefit from a stronger engagement with prior research. It currently lacks critical comparisons with existing studies, which weakens the findings of this study. Incorporating relevant prior findings and discussing how the results align with or deviate from them would help contextualize the study’s contribution and explain how it advances current knowledge in the field.*

**Response 6:**

We sincerely appreciate the reviewer’s insightful comment. Considering the specific characteristics of the dataset and the study region, we have conducted a comparative analysis between our proposed framework—which classifies rainfall into four detailed categories (light, moderate, heavy, and very heavy)—and a reference framework adapted from Hirose et al. (2019) [5]. The latter employs a two-class classification scheme (light rain vs. heavy rain) with a threshold of 1.8 mm/h. This analysis has been added to the revised manuscript in **Section 3.4.2 Impact of multi-classes classification.** Also, the comparaed study, Hirose et al. (2019) [5], has been added to Section **Reference**

**Point 7:** *Finally, the conclusion section should be revised for brevity and focus. It should not repeat detailed findings but instead synthesize the main contributions, outline their implications, and briefly note directions for future work.*

**Response 7:**

We sincerely thank the reviewer for this insightful and accurate comment. Accordingly, we have revised the Conclusion section to be more concise and focused. These updates have been incorporated into **Section 4. Conclusion**.
