# Supplementary material for: Enhancing rainfall estimation accuracy with machine learning, cloud masking, and multi-source data: A case study of four coastal provinces in central Vietnam
Source: PLoS One. 2026 Feb 13;21(2):e0342825. doi: 10.1371/journal.pone.0342825 (PMC12904594; doi:10.1371/journal.pone.0342825)
Supplement: Attachment — Submitted filename: Response to Reviewers.docx [file pone.0342825.s002.docx]

**Responses to reviewers comments**

**Manuscript Title**: **Enhancing Rainfall Estimation Accuracy with Machine Learning, Cloud Masking, and Multi-Source Data: A Case Study of Four Coastal Provinces in Central Vietnam**

**Manuscript ID**: PONE-D-25-46623

Dear Editor and Reviewers,

We would like to express our sincere thanks to the editor and anonymous reviewers for their time spent in evaluating our manuscript and providing us with various constructive comments that help us to improve the presentation of our manuscript. We have carefully addressed all the editor and reviewer’s comments and provided a point-by-point reply. The comments of Reviewers are typeset in normal blue color italic font, and our responses are written in black font color. Additionally, in the revised manuscript, explanations and justifications according to reviewer’s comments have been highlighted in red font color. Other minor corrections have not been highlighted.

Yours sincerely,

Authors.

**1. Response to Editor**

*Please review your reference list to ensure that it is complete and correct. If you have cited papers that have been retracted, please include the rationale for doing so in the manuscript text, or remove these references and replace them with relevant current references. Any changes to the reference list should be mentioned in the rebuttal letter that accompanies your revised manuscript. If you need to cite a retracted article, indicate the article’s retracted status in the References list and also include a citation and full reference for the retraction notice.*

**Response :**

We appreciate the Editor’s important reminder regarding the reference list. We have conducted a comprehensive review of all cited works and would like to report the following adjustment:

1. Modification of the ***Reference*** in the revised manuscript: To ensure strict adherence to the journal’s formatting standards and professional style, we have decided to remove the original reference *[31]: T. Cong TT, et al., “The application of himawari satellite data in forecast and warning of rain and thunderstorm*”, Vietnam J Hydrometeorol, 2020. doi: 10.36335/VNJHM.2020(719).1–13. The rationale for this removal is that the article is published exclusively in Vietnamese (under the title “*Ứng dụng số liệu ảnh mây vệ tinh Himawari trong dự báo và cảnh báo mưa dông cho khu vực Đồng bằng sông Cửu Long*”) and lacks an official English version. We certify that the exclusion of this reference does not affect the evaluation or interpretation of the research results presented in our manuscript. Following the removal of Reference [31], we have updated the citation numbering accordingly, and these changes have been highlighted in yellow in the revised manuscript.

2. Citation renumbering in the text: Consequently, we have updated the in-text citations in the "**Mask cloud generation**" section. The original citation sequence [29], [30], [31], [32] has been revised to [29], [30], [31].

The new reference [31] is now: Y. Jing et al., “Characteristics of Summer Hailstorms Observed by Radar and Himawari-8 in Beijing, China,” Remote Sens (Basel), vol. 14, p. 5843, Jun. 2022, doi: 10.3390/rs14225843.

3. Confirmation: The References section has been updated accordingly (the total number of references has decreased by one). We confirm that all remaining references are accurate, properly formatted according to PLOS ONE guidelines, and strictly adhere to publication ethics (no retracted papers are cited).

**2. Response to Reviewers**

**A. Reviewer 1**

*The potential lack of relevance for extreme events reflects a deliberate methodological choice to optimize global error. However, it should be noted that high-intensity, rare, or localized events could still substantially influence model outcomes.*

**Response to Reviewer 1:**

We sincerely thank the reviewer for this insightful observation. We acknowledge that the proposed model is primarily optimized for global performance metrics, which may reduce its responsiveness to rare or extreme rainfall events. At the same time, we recognize that high-intensity or localized rainfall can still exert a meaningful influence on model outcomes, particularly in short-term or event-focused applications, or when such events are underrepresented in the training dataset. A more detailed investigation of these high-intensity events will therefore be considered in future work.

Therefore, in the revised manuscript, we have revised the final paragraph of the **Conclusion** section to explicitly acknowledge this limitation:

“Furthermore, the architecture’s design establishes a robust and flexible methodological foundation that allows for future development into near real-time applications through the straightforward substitution of reanalysis data with real-time meteorological features. As the framework is extended to such applications, rare and extreme rainfall events may still influence model performance, particularly in short-term or event-based contexts, highlighting the need for a more detailed assessment of high-intensity events in future work.”

**B. Reviewer 2**

*The authors have addressed the reviewers’ comments and made the necessary corrections. However, the manuscript should be carefully proofread prior to publication, and the quality of the figures should be further improved to enhance clarity and readability.*

**Response to Reviewer 2:**

We sincerely thank the reviewer for your valuable and constructive feedback. We have thoroughly reviewed and revised the manuscript in accordance with your suggestions.

1. Language and proofreading: We have carefully proofread the entire manuscript to correct typographical and grammatical errors. All modifications have been highlighted in yellow in the revised manuscript to facilitate the review process.

2. Figure quality: We have comprehensively improved the quality of the figures to enhance both resolution and readability. We have standardized the presentation by increasing the font size of axis labels, tick marks, and legends across all figures. The specific improvements for each figure are detailed below:

Figure 2: We increased the font size and applied bold formatting to the numerical values to significantly improve legibility.

Figure 3: We increased the font size and bolded the lines and arrows. Furthermore, the image resolution was enhanced from 300 dpi to 400 dpi.

Figure 4: We increased the font size for text and numbers and bolded both the horizontal and vertical axis labels.

Figures 5 and 7: We increased the font size and bolded the axis labels. Additionally, we changed the background color from purple to blue to improve contrast and visual clarity.

Figures 6, 8, 9, 11, and 13: We increased the font size for all text and numerical values to ensure they are clearly legible.

Figure 10: We increased the font size and bolded the axis labels. The resolution was also enhanced from 300 dpi to 400 dpi.

Figure 12: We increased the font size and enlarged the overall dimensions of the figure.

Figure 14: We increased the font size, bolded the axis labels, and enlarged the overall dimensions of the figure.
